# Evaluating the fragility and robustness of randomized controlled trials in proximal humerus fracture management

**DOI:** 10.1016/j.jseint.2025.09.002

**Published:** 2025-09-17

**Authors:** Matthew T. McKinley, Aghdas Movassaghi, Chase Burzynski, Lana Smith, Guoli Zhou, Justin T. Childers, Jocelyn Lubert, Garrett R. Jackson, Vani J. Sabesan

**Affiliations:** a Nova Southeastern University Dr. Kiran C. Patel College of Osteopathic Medicine, Davie, FL, USA; b Michigan State University College of Human Medicine, East Lansing, MI, USA; c University of Georgia, Athens, GA, USA; d Charles E. Schmidt College of Medicine, Florida Atlantic University, Boca Raton, FL, USA; e Orthopaedic Center of Palm Beach County, Atlantis, FL, USA; f University of Missouri, Department of Orthopaedic Surgery, Columbia, MO, USA

**Keywords:** Proximal humerus fracture, Randomized controlled trial, Systematic review, Fragility index, Continuous fragility index, Loss to follow-up

## Abstract

**Background:**

Despite multiple surgical techniques for proximal humerus fractures (PHFs), no method has demonstrated superiority, and randomized controlled trials (RCTs) on PHF treatments have yielded conflicting results. The purpose of this study is to analyze the fragility indices for treatment of PHF by calculating fragility index (FI) and continuous fragility index (CFI) for PHF trials outcomes.

**Methods:**

A systematic review was conducted following Preferred Reporting Items for Systematic Reviews and Meta-Analyses to evaluate PHF RCTs. RCTs reporting at least one statistically significant dichotomous outcome published between 2000 and 2024 were included. The FI and CFI for continuous outcomes were calculated for each trial.

**Results:**

Seventeen RCTs were included in FI analysis. The FI was 1 across 21 outcomes, meaning it would take 1 patient reversal to alter significance (FI: 1, interquartile range 0-2.5). Ten RCTs were included in the CFI analysis, which had a median of 4, indicating that across these 10 RCTs, changing 4 points on patient outcomes could nullify statistical significance (CFI: 4, interquartile range 0-8.0). After adjustment, the reported *P* value remained an independent predictor of fragility, with an effect size of β = −166.7 (standard error = 51.6, *P* = .0103) for CFI.

**Conclusion:**

The observed median FI and CFI suggest significant findings of PHF RCTs could be modified with a single patient switching groups or patient-reported outcomes by changing just 4 points. Surgeons need to be skeptical of the results and conclusions drawn from high-level studies and consider the fragility and significant loss of follow-up impacting conclusions.

Proximal humerus fractures (PHFs) are a significant health concern, especially among individuals older than 50 years, ranking as the third most frequent fragility fracture.^20^^,^^39^ There are several treatment options available for PHF, including nonoperative treatment, closed reduction and percutaneous pinning, open reduction and internal fixation (ORIF), ORIF with an intramedullary nailing (IMN), reverse total shoulder arthroplasty (rTSA), and hemiarthroplasty (HA).^5^ It has been reported that nonoperative treatment is the most commonly used approach, whereas ORIF and rTSA are frequently selected as surgical options for more complex cases.^33^ Despite the availability of multiple surgical techniques, no single method has been consistently proven superior, and randomized controlled trials (RCTs) on PHF treatments have yielded conflicting results.^11^^,^^16^^,^^19^^,^^28^^,^^46^ RCTs often suffer from variable statistical power, resulting in fragile findings that may not provide reliable guidance for clinical decision-making.^31^^,^^36^

To address these challenges, the fragility index (FI) was developed as a complementary measure to the *P* value, assessing the robustness of RCT findings.^12^^,^^44^ The FI quantifies how many events would need to be altered to nonevents to shift a significant finding to becoming a nonsignificant one, providing a clearer perspective on the stability of the evidence.^42^ While some review studies, such as those by Caroll et al and Kyriakides et al, have applied the FI to PHF outcomes, they primarily focus on secondary outcomes, such as complications, rather than clinical outcomes and function.^9^^,^^26^

Recent advancements have proposed additional metrics to assess RCT robustness more comprehensively. These include the fragility quotient (FQ), continuous fragility index (CFI), and continuous fragility quotient (CFQ), which extend fragility analysis to continuous outcome measures. Metrics like the CFI are particularly relevant for evaluating objective and subjective continuous outcome measures such as the American Shoulder and Elbow Surgeons (ASES) score, Constant Score (CS), Disabilities of the Arm, Shoulder, and Hand (DASH) score, Neer score, and visual analog scale.^2^^,^^4^

Given the limitations of current RCTs and the lack of consensus on the best surgical approach for PHF, this study aimed to provide a comprehensive analysis of the fragility indices by calculating the FI, FQ, CFI, and CFQ for PHF trial outcomes. It is hypothesized that the number of event outcome reversals needed to alter the significance of the study would be fewer than the number of patients lost to follow-up for a majority of the studies included in the analysis.

## Methods

### Search strategy and study selection

A systematic review of the literature was performed following the 2020 Preferred Reporting Items for Systematic Reviews and Meta-Analyses guidelines.^32^ The computerized databases PubMED, Embase, and the Cochrane library were searched for RCTs published between January 2000 and June 2024 using the following search terms: ("proximal humerus fracture") AND ("treatment" OR "surgery") AND ("randomized controlled trial" OR "randomized controlled trials" OR "RCT").

The inclusion criteria limited the search to papers with level I and II evidence published in English. Eligible studies included original RCTs with 2 parallel study arms using a one-to-one allocation of human subjects to either treatment or control groups that report at least one significant dichotomous outcome (*P* < .05). Exclusion criteria included non-English publications, biomechanical studies, review articles, cadaveric studies, nonrandomized and cohort studies, case-control studies, animal studies, abstracts, cross-sectional studies, case reports, editorials, commentaries, protocols, and retrospective studies.

Following identification of studies with significant dichotomous outcomes that met the inclusion criteria above, further inclusion criteria was applied to develop a CFI. The inclusion criteria for CFI were as follows: (1) the study must be an RCT about PHF treatments; (2) it must involve only 2 treatment arms (control vs. intervention); (3) the study found at least one statistically significant continuous outcome between the compared groups at any time during the study; (4) the study reports sample size, mean, and a measure of variability (standard deviation, confidence intervals, interquartile range (IQR), etc.) for both treatment and control arms. Two independent reviewers screened titles and abstracts, and a third reviewer resolved discrepancies.

### Data extraction

Data from the selected articles were extracted and placed into an Excel spreadsheet (Microsoft Excel, version 16.70; Microsoft Corporation, Redmond, WA, USA) for analysis. The following data were abstracted from the included studies: title, journal, author(s), year of publication, clinical trial registration status, multicenter vs. single-center design, randomization method, allocation method, intervention and control sample definitions, total sample size of studies, total number of patients lost to follow-up in each study, mean follow-up time, primary outcomes, secondary outcomes, number of events in the intervention and control groups for all statistically significant dichotomous outcomes, and JADAD score. Studies were evaluated for methodological quality independently by 2 reviewers using the JADAD scale, where a score of greater than 3 indicated a reduced to no risk of bias and a score lower than 3 indicated a moderate to higher risk of bias.^21^

### Fragility index calculation

For all statistically significant dichotomous variables, the FI was calculated by repeatedly applying Fisher exact test, moving one individual at a time from the events group to the nonevents group until the dichotomous variable was no longer significant (*P* > .05). An FI of 1 indicates that shifting a single individual from the event group to the nonevent group would render the result insignificant. Smaller FI values indicate greater fragility, as fewer changes are required to overturn significance. All FI calculations were performed using an online Fischer exact test calculator, https://www.graphpad.com/quickcalcs/contingency1/.

### Continuous fragility index calculation

The CFI measures how many patient outcomes need to change to make a continuous, statistically significant result no longer significant. The CFI of each continuous outcome for individual studies was calculated using an online calculator (https://jmcaldwell.shinyapps.io/CFIApp/)^7^ that applies an iterative substitution algorithm to estimate fragility in continuous outcomes. Since full raw data sets are often unavailable in RCTs, the calculator generates normally distributed data sets based on reported means, standard deviations, and sample sizes. A Welch t-test is performed iteratively, shifting data points from the higher-mean group to the lower-mean group until the *P* value exceeds .05, indicating a nonsignificant difference. The CFI represents the number of iterations required to reach this threshold. Default settings for parameters, including a tolerance of 0.01 and one iteration, were used due to unavailable raw data from each included study. From the calculated CFI, a CFQ was calculated for each study. The FQ and CFQ were calculated by dividing the FI and CFI by the sample size of the study. These metrics standardize each trial's fragility relative to its sample size, reducing potential bias.^42^

### Statistical analysis

Descriptive statistics were used to summarize the CFI and CFQ for eligible RCTs. Categorical variables were presented as numbers and percentages, while continuous variables were summarized using the median and IQR. Bivariate associations between individual contributing factors (study characteristics) and CFI/CFQ were analyzed with a Kruskal–Wallis test and Pearson correlation analysis. Multivariate analysis was conducted to examine the association between potential contributing factors using rank-based estimation regression model.^23^ All data management and analyses were conducted in R-4.4.1 (R Core Team, 2021; R Foundation for Statistical Computing, Vienna, Austria).^1^

## Results

**Figure 1:**
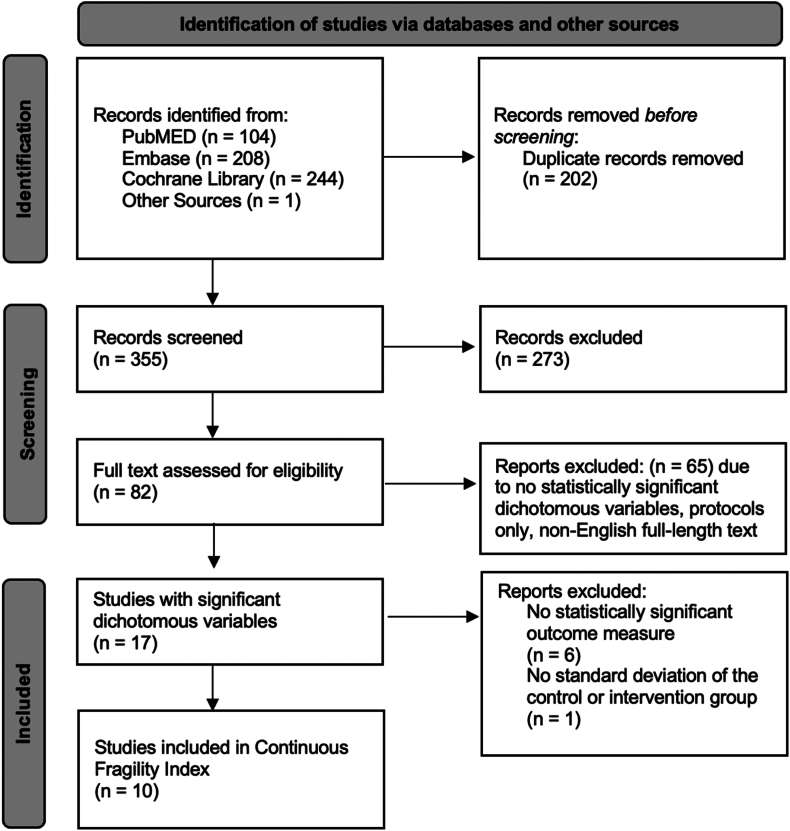
PRISMA flow diagram for studies reporting on proximal humerus fracture treatments. *PRISMA*, Preferred Reporting Items for Systematic Reviews and Meta-Analyses.

The comprehensive search identified a total of 557 studies, with 355 studies remaining following the removal of duplicates. Of the 82 studies that underwent full-text screening, 17 (1,033 patients) met the inclusion criteria to be included in the initial FI analysis. Of these, 6 RCTs lacked statistically significant findings for a continuous outcome measure, and 1 did not report the standard deviation for either the control or intervention group. As a result, 10 studies met the criteria for CFI calculation (Fig. 1). Of all included studies, 47% (8/17) were found to have a JADAD score of less than 3.

### Fragility index

The median number of patients randomized per study in FI analysis was 61 (IQR: 34-96), and the median proportion of patients lost to follow-up was 4 (IQR: 0-18). The studies had an average follow-up length of 24 months (IQR: 15.6-30.8 mon). Eight of the 17 studies were level I evidence (47%), and 9 were level II evidence (53%). The studies were characterized by the following interventions for proximal humerus treatment: locking plate fixation (6 studies, 35.3%), IMN (5 studies, 29.4%), HA (3 studies, 17.6%), nonoperative treatment (2 studies, 11.8%), and rTSA (1 study, 5.9%).

**Figure 2:**
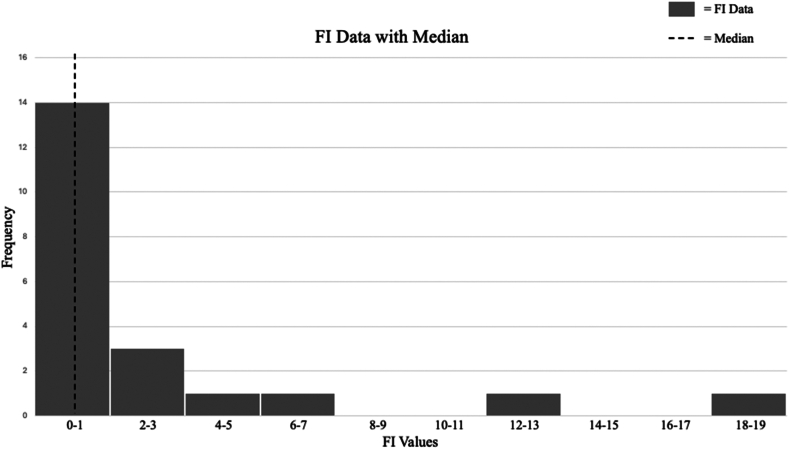
Histogram of study-specific FI values. *FI*, fragility index.

Twenty-one statistically significant outcomes were extracted from the 17 included studies to be calculated for the FI. Fig. 2 is a histogram of the FI for all analyzed outcomes. The median FI was 1 (IQR: 0.0-2.5), indicating that in half of the included studies, reversing the outcome of a single patient would be sufficient to render the result statistically nonsignificant. The median FQ of noncontinuous outcomes was 0.029 (IQR: 0.000-0.048). Seven of the 21 significant dichotomous outcomes had an FI of 0 (33%), while 9 out of 21 had more patients lost to follow-up than their FI (43%) (Table I), demonstrating extreme fragility.

**Table I:** FI and FQ, lost to follow-up, and *P* values for each outcome included in the analysis of noncontinuous outcomes.

Study	Intervention	Control	Dichotomous, significant outcome	Sample size	JADAD score	FQ	FI	LTFU	*P* value
Zhu,^48^ 2011	Locking IMN	Locking plates	Complications	51	3+	0.020	1	6	.024
Zhang,^47^ 2011	Locking plate + medial support screws group	Locking plate only	Failure rate medial support screws	68	3+	0.015	1	4	.036
Liu,^29^ 2011	Locking plate + minimally invasive injectable graft	Locking plate only	Complications	50	<3	0.020	1	0	<.05
Soliman,^41^ 2013	Hemiarthroplasty and tenodesis of LHB	Hemiarthroplasty	Shoulder pain	37	3+	0.000	0	0	.03
Sebastia-Forcada,^39^ 2014	Reverse shoulder arthroplasty	Hemiarthroplasty	Revision survival	61	3+	0.000	0	0	.043
Lopiz,^30^ 2014	Curvilinear nail	Straight nail	Rotator cuff disease symptoms	52	3+	0.058	3	2	.001
Lopiz,^30^ 2014	Curvilinear nail	Straight nail	Proximal screw backout	52	3+	0.038	2	2	.02
Jin,^22^ 2016	Locking plate + probing method with depth gauge	Locking plate traditional measuring method	Screw penetration,	40	<3	0.025	1	0	.027
Gracitelli,^15^ 2016	IMN	Locking plate	Complications	65	3+	0.185	12	4	.001
Gracitelli,^15^ 2016	IMN	Locking plate	Reoperation rate	65	3+	0.000	0	4	.041
Gracitelli,^15^ 2016	IMN	Locking plate	Hardware problems	65	3+	0.000	0	4	.041
Chen,^10^ 2016	Hemiarthroplasty	Locking plate	Subjective excellent ratings from patients	56	<3	0.107	6	4	<.05
Peng,^34^ 2017	Locking plate + biomimetic mineralized collagen putty	Locking plate	Total complications	80	<3	0.000	0	0	<.05
Peng,^34^ 2017	Locking plate + biomimetic mineralized collagen putty	Locking plate	Excellent/good rate	80	<3	0.000	0	0	<.05
Carbone,^8^ 2017	Immediate intensive mobilization program	Immediate conventional mobilization	Noncompliant patients to rehabilitation program	80	3+	0.000	0	4	.037
Plath^35^ 2019	Locking blade nail	Locking plate	Secondary loss of reduction and screw cut-out	68	<3	0.029	2	13	.039
Rouleau,^37^ 2020	Locking plate + deltoid split approach	Locking plate with standard deltopectoral approach	Axillary nerve identification	85	3+	0.212	18	16	<.0001
Zhang,^46^ 2020	Small splint + drugs	Locking plate	Complications	86	<3	0.012	1	0	.026
Boyer,^6^ 2021	IMN	Locking plate	Complications	85	<3	0.059	5	9	.003
Karslioglu,^24^ 2024	Locking plate + vascularized pectoralis major bone graft	Locking plate with iliac crest bone graft	Reduction loss	34	<3	0.029	1	0	.013
Fialka,^14^ 2008	Hemiarthroplasty with EPOCA Implant	Hemiarthroplasty with HAS implant	Tuberosity resorption	35	3+	0.029	1	5	<.05

*IMN*, intramedullary nailing; *LHB*, long head of the biceps; *FQ*, fragility quotient; *FI*, fragility index; *LTFU*, lost to follow-up; *HAS*, hemiarthroplasty system.

### Continuous fragility index

The 10 studies included in the calculation of the CFI comprised of 631 total patients, with a median of 32.0 patients in the control group (IQR: 17-43) and a median of 30.0 patients in the intervention group (IQR: 17-43). The median proportion of patients lost to follow-up was 4.50 (IQR: 0-16). Six of the included studies were level II evidence (60%), while 4 were level I evidence (40%).

**Figure 3:**
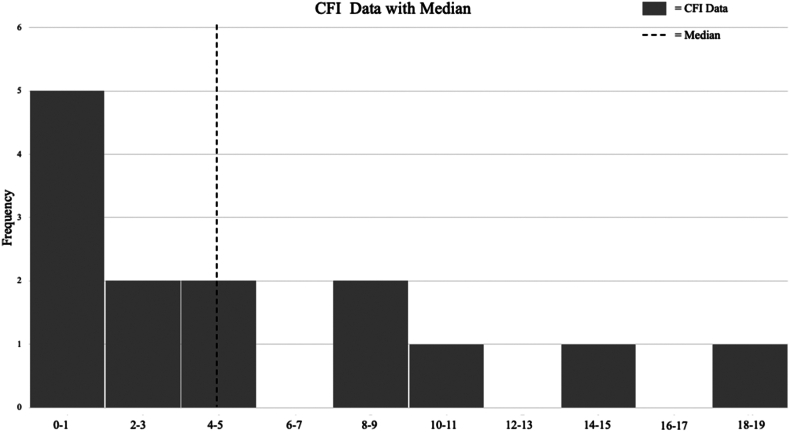
Histogram of study-specific CFI values. *CFI*, continuous fragility index.

Fourteen statistically significant subjective continuous outcomes were extracted from the 10 included studies to be calculated for the CFI. Fig. 3 is a histogram of the CFI for all analyzed outcomes. The median CFI for subjective outcomes was 4 (IQR: 0.0-8.0) The median CFQ of continuous outcomes was 0.081 (IQR: 0.000-0.116). Four of the 14 CFIs were zero (29%), and 8 out of 14 CFIs had lost to follow-up greater than the CFI (57.1%) (Table II). For these outcomes, loss to follow-up was on average 2.91 times greater than the CFI; ie, 2.91 more patients were lost to follow-up than the number of patients needed to be moved to achieve nonsignificance.

**Table II:** CFI, CFQ, lost to follow-up, and *P* values for each outcome included in the analysis of continuous outcomes.

Study	Intervention	Control	Outcome measure	JADAD score	Sample size	CFQ	CFI	LTFU	*P* value
Boyer,^6^ 2021	IMN	Locking plate	ASES	<3	85	0.165	14	9	.001
Peng,^34^ 2017	Locking plate+	Locking plate	ASES	<3	80	0.238	19	0	.049
Karslioglu,^24^ 2024	Locking plate+	Locking plate	ASES	<3	34	0.088	3	0	.007
Boyer,^6^ 2021	IMN	Locking plate	CS	<3	85	0.000	0	9	.043
Chen,^10^ 2016	Hemiarthroplasty	Locking plate	CS	<3	60	0.033	2	4	.049
Plath,^35^ 2019	IMN	Locking plate	DASH	<3	55	0.000	0	13	.042
Chen,^10^ 2016	Hemiarthroplasty	Locking plate	DASH	<3	60	0.000	0	4	.049
Boyer,^6^ 2021	IMN	Locking plate	VAS	<3	85	0.094	8	9	.001
Zhang,^46^ 2020	Small Splint + drugs	Locking plate	VAS	<3	86	0.128	11	0	.001
Zhu,^48^ 2011	IMN	Locking plate	ASES	3+	57	0.088	5	6	.021
Zhu,^48^ 2011	IMN	Locking plate	VAS	3+	57	0.000	0	6	.042
Zhang,^46^ 2011	Locking plate+	Locking plate	CS	3+	68	0.074	5	4	.01
Soliman,^41^ 2013	Hemiarthroplasty	Hemiarthroplasty	CS	3+	37	0.027	1	0	.04
Rouleau,^37^ 2020	Locking plate+	Locking plate	DASH	3+	69	0.116	8	16	.003

*IMN*, intrameduallry nailing; *ASES*, American Shoulder and Elbow Surgeons; *CS*, Constant Score; *DASH*, Disabilities of the Arm, Shoulder and Hand; *VAS*, visual analog scale; *CFQ*, continuous fragility quotient; *CFI*, continuous fragility index; *LTFU*, lost to follow-up.

Grouped by proximal humerus treatment intervention, the median CFI values were as follows: HA, 1.0 (IQR: 1.0); IMN, 2.5 (IQR: 7.2); locking plate, 6.5 (IQR: 6.2); and small splint with pharmacologic treatment, 11.0 (IQR: 0.0). The corresponding median CFQ values were HA, 0.03 (IQR: 0.02); IMN, 0.04 (IQR: 0.04); locking plate, 0.10 (IQR: 0.06); and small splint with pharmacologic treatment, 0.06 (IQR: 0.06) (Table III). To explore whether fragility varied by intervention type, we grouped studies descriptively by treatment arm. The median CFI was 9.5 (IQR: 10.7) for the ASES score, 1.5 (IQR: 2.0) for the CS, 0.0 (IQR: 4.0) for the DASH score, and 8.0 (IQR: 5.5) for visual analog scale score. Although numerical differences were observed, a Kruskal–Wallis test revealed no statistically significant difference in median CFI between treatment types (*P* = .2091). (Table III).

**Table III:** Frequency distribution of the fragility index/quotient across all potential categorical contributing factors (N = 14).

	Fragility index			Fragility quotient		
	Median (IQR)	W or chi∗	*P* value	Median (IQR)	W or chi	*P* value
JADAD score:						
≥3	5.0 (4.0)	20.00	.787	0.07 (0.06)	18.50	.637
<3	2.5 (10.2)			0.06 (0.12)		
Intervention:						
Hemiarthroplasty	1.0 (1.0)	4.5363	.2091	0.03 (0.02)	4.9395	.1763
IMN	2.5 (7.2)			0.04 (0.04)		
Locking plate+	6.5 (6.2)			0.10 (0.06)		
Small splint + drugs	11.0 (0.0)			0.06 (0.06)		
Control:						
Hemiarthroplasty	1.0 (0.0)	4.00	.6151	0.03 (0.00)	4.00	.6159
Locking plate	4.0 (8.0)			0.08 (0.11)		
Primary outcome:						
ASES	9.5 (10.7)	4.3125	.2296	0.13 (0.09)	5.0819	.1659
CS	1.5 (2.0)			0.03 (0.02)		
DASH	0.0 (4.0)			0.00 (0.06)		
VAS	8.0 (5.5)			0.09 (0.06)		
Benefiting from intervention:						
No	8.0 (0.0)	0.5759	.7498	0.12 (0.00)	0.78876	.6741
Noninferior	4.0 (9.5)			0.05 (0.11)		
Yes	2.5 (4.7)			0.05 (0.08)		

*IQR*, interquartile range; *ASES*, American Shoulder and Elbow Surgeons; *CS*, Constant Score; *DASH*, Disabilities of Arm, Shoulder, and Hand; *VAS*, visual analog scale; *IMN*, intermeduallry nailing.

∗ W, Wilcoxon rank-sum test for comparison between 2 groups; Chi, Kruskal–Wallis chi-squared from Kruskal–Wallis rank-sum test for comparison among at least 3 groups.

Pearson correlation analysis demonstrated that among continuous contributing factors, only the *P* value reported in the included studies showed a statistically significant association with both the CFI and CFQ. A moderate negative correlation was observed (r = −0.55, *P* = .04 for CFI and r = −0.55, *P* = .04 for CFQ), indicating that studies with lower *P* values were associated with greater fragility (Table IV). No other continuous study factors showed a significant correlation with CFI or CFQ (*P* > .05). Further nonparametric linear regression analysis was performed to evaluate whether the association between reported *P* value and fragility remained significant after adjusting for continuous subjective outcomes. After adjustment, the reported *P* value remained an independent predictor of fragility, with an effect size β = −166.7 (standard error = 51.6, *P* = .0103) for CFI and β = −2.01 (standard error = 0.48, *P* = .0024) for CFQ. Even after adjusting for primary outcome measures, studies with lower *P* values are more fragile and need fewer event reversals to lose statistical significance.

**Table IV:** Correlations between the continuous outcome variable fragility index/quotient and the individual continuous contributing factors (N = 14).

Contributing factors	Fragility index		Fragility quotient	
	Correlation coefficient (r)∗	*P* value	Correlation Coefficient (r)	*P* value
N. control	0.529	.0516	0.456	.1012
Mean. control	0.176	.5474	0.178	.5431
Sd. control	−0.227	.4347	−0.267	.3567
N. intervention	0.519	.0572	0.47	.0901
Mean. intervention	0.222	.444	0.231	.4266
Sd. intervention	0.106	.716	0.089	.7625
*P* value	−0.546	.0434	−0.553	.0404
Dropout	−0.143	.6249	−0.174	.5527
Complications. intervention	−0.101	.7314	−0.026	.9296
Complications control	0.218	.4541	0.23	.4293
Tot. complications	0.073	.8045	0.123	.6752

∗ Spearman rank correlation.

## Discussion

This study highlights the statistical fragility of RCT outcomes for PHF treatments, as assessed by both continuous and noncontinuous fragility metrics. Among the 17 studies with significant dichotomous outcomes, the median FI was 1, and the median FQ was 0.029, indicating that statistical significance could be lost with a single event change. For continuous outcomes, the median CFI was 4, and the median CFQ was 0.081, indicating that shifting just 4 points on patient outcomes could nullify statistical significance.

Previous studies have evaluated the FI of dichotomous outcomes of several PHF treatment options and shoulder and elbow surgery more broadly.^9^^,^^26^^,^^40^ Compared to those studies, which calculated median FIs of 1 and 4 for dichotomous outcomes, this study expands the analysis to include primary continuous subjective outcome measures.^9^^,^^26^^,^^40^ Although our study investigated continuous measures, it continues to follow the trend of the 2 PHF treatment fragility studies prior to ours, in that all studies have quite high rates for follow-up losses exceeding each FI. Carroll et al and Kyriakides et al, who assessed dichotomous outcomes for PHF, reported rates of 50% and 41.1% for losses exceeding their FIs, respectively, and the present study found 57.1%.^9^^,^^26^ Despite assessing different aspects of fragility, each study that looks at PHF treatments found many patients are lost to follow-up, which could alter the significance of the findings.

Loss to follow-up was a key factor influencing trial fragility. In our study, 57.1% of continuous outcomes had follow-up losses exceeding their CFI, a rate higher than previously reported.^4^^,^^17^^,^^18^ Al-asadi et al found that 38.6% of anterior shoulder instability trials had follow-up losses exceeding their CFI, while Carroll et al, Kyriakides et al, and Ruzbarsky et al reported rates of 50%, 41.1%, and 87.5%, respectively, for significant dichotomous outcomes in shoulder and elbow surgery trials.^4^^,^^9^^,^^26^^,^^38^ When follow-up losses exceed the number of events required to overturn statistical significance, missing data could significantly alter results and conclusions from these studies. Since patients lost to follow-up often have worse outcomes, their exclusion may introduce bias. While all analyzed outcomes were reported as statistically significant in their respective trials, the wide variability in FI and CFI values suggest differing levels of robustness. This variability underscores the need for caution while interpreting RCT data, as statistically significant findings should not all carry the same weight in clinical decision-making.

The lowest CFI was observed in HA trials (median CFI = 1, IQR: 1). Prior systematic reviews have reported inconsistent outcomes for HA in PHFs.^19^^,^^28^ In our study, the DASH and CS scores had a CFI of zero or close to zero, indicating that their reported statistical significance was highly unstable and could be lost with minimal data changes. Compared to RCTs in other orthopedic subspecialties, PHF trials exhibited some of the lowest CFI and CFQ values, reinforcing concerns about their fragility (Table V). However, our study did find the ASES score (median = 9.5, IQR = 10.7) to be the most robust outcome score. Surgeons may rely more on ASES in RCTs, as it has been found to be the most reliable and robust patient-reported outcome for this population of PHF patients.

**Table V:** CFI and CFQ for orthopedic specialties.

Study	Specialty	Median CFI	Median CFQ
Current study	Shoulder and elbow	4.0	0.081
Caldwell/Kahn,^7^^,^^25^ 2021	Sports medicine	7.0	NR
Gupta,^17^ 2022	Spine/neurosurgery	3.5	0.070
Gupta,^18^ 2022	Foot and ankle	5.0	0.122
Xu,^45^ 2022	Foot and ankle	4.0	0.105
Lamiere,^27^ 2024	Total joint	5.7	0.131
Al-Asadi,^4^ 2024	Shoulder and elbow	8.2	NR
Ahn,^3^ 2024	Foot and ankle	8.8	0.100
Dworsky-Fried,^13^ 2024	Sports medicine	4.9	NR
Abesteh,^2^ 2025	Shoulder and elbow	5.85	NR
Villarreal-Espinosa,^43^ 2025	Sports medicine	8.0	NR

*NR*, not reported; *CFI*, continuous fragility index; *CFQ*, continuous fragility quotient.

While FI and CFI cannot be directly compared, evaluating both provides a more comprehensive assessment of trial robustness. Zhu et al reported an FI of 1 for complications, yet the CFI for ASES was 5, indicating a more stable continuous outcome.^49^ Similarly, Peng et al had an FI of 0 for total complications but a high CFI for ASES, suggesting that dichotomous outcomes alone may overstate fragility.^34^ These findings support incorporating both FI and CFI analyses in RCT appraisal, as many orthopedic trials report continuous outcome measures in addition to dichotomous endpoints, and more frequently, success is determined by continuous outcomes of RCTs.

It is important to note that while these results reveal the fragility of PHF RCTs, they do not invalidate the statistical significance of these trials. Instead, the findings highlight the importance of interpreting statistical significance cautiously, particularly in the context of loss to follow-up and suggest RCT methodology could be improved. Nearly half of included studies had a JADAD score < 3, suggesting future trials should prioritize improved randomization, blinding, and outcome reporting to reduce risk of bias.

It is recommended to use CFI and FI together as supplementary tools, alongside follow-up data, to provide a more comprehensive understanding of trial robustness. Improved follow-up protocols in PHF RCTs will be critical to ensuring more reliable and meaningful results in future studies. In addition, it is suggested that surgeons can rely on their clinical expertise and familiarity with specific procedures to tailor treatment to individual patient needs for PHF treatment, as RCT findings are fragile for both dichotomous and continuous outcomes.

This study has several limitations. The CFI was calculated only for studies eligible for an FI analysis, requiring a statistically significant dichotomous outcome. This excluded several trials—including some evaluating more commonly used interventions, such as rTSA and nonoperative management—that may have otherwise contributed meaningful insight into fragility. However, setting the inclusion and exclusion criteria as it was in the present study allowed for more articles to be included in FI than previous FI analyses and allowed for methodological consistency between previous studies, while introducing the novel CFI to PHF literature. Taken together, the FI and CFI allow for an assessment of trial robustness that neither would be able to capture alone. While studies may have been excluded from CFI investigating more common treatments, the present study is still able to conclude that, systemically, RCTs are fragile among the various treatments for PHF. Although subgroup differences in CFI were observed, these findings should be interpreted cautiously given the limited number of studies and variability in study design. In addition, nearly half of included studies had JADAD scores < 3, indicating methodological limitations that may contribute to fragility regardless of intervention type. In addition, reliance on reported descriptive statistics rather than raw outcome data introduced an assumption of normal distribution, which may not hold for all datasets. The impact of this was reduced by using Kruskal–Wallis and nonparametric logistic regression, which do not require normality assumptions. Lastly, high rates of patient loss to follow-up in the included studies also limit data reliability. Standardized follow-up protocols in clinical trials could improve retention and data completeness. Despite these limitations, this study provides important insights into the fragility of PHF literature and allows for FI and CFI to be analyzed alongside each other.

## Conclusion

This study demonstrates that randomized trials evaluating PHF treatments are highly fragile. With a median FI of 1 and CFI of 4, many statistically significant findings could be reversed by a single event change or minimal shift in patient-reported outcomes. These results are consistent with prior FI and CFI studies in orthopedics and reinforce the need for cautious interpretation, particularly in trials with substantial loss to follow-up.

## Disclaimers:

Funding: No funding was received for this article.

Conflicts of interest: The authors, their immediate families, and any research foundation with which they are affiliated have not received any financial payments or other benefits from any commercial entity related to the subject of this article.
